# Screening and management of portal hypertension and varices in cirrhosis: Expert perspectives

**DOI:** 10.1097/HC9.0000000000000682

**Published:** 2025-04-03

**Authors:** Robert S. Brown, Kimberly A. Brown, Steve Flamm, Rachel E. Bejarano, Robert S. Rahimi, Ashwani K. Singal, Don C. Rockey

**Affiliations:** 1 Department of Medicine, Division of Gastroenterology and Hepatology, Weill Cornell Medicine, New York, New York, USA; 2 Department of Medicine, Division of Gastroenterology and Hepatology, Henry Ford Health, Detroit, Michigan, USA; 3 Division of Gastroenterology and Hepatology, Northwestern University Feinberg School of Medicine, Chicago, Illinois, USA; 4 The Chronic Liver Disease Foundation, Clark, New Jersey, USA; 5 Baylor University Medical Center; Texas A&M Health Science Center—College of Medicine; Baylor Scott & White Liver Consultants of Texas—Dallas; Charles A. Sammons Cancer Center, Dallas, Texas, USA; 6 Division of Gastroenterology and Hepatology, Louisville School of Medicine, Louisville, Kentucky, USA; 7 Digestive Disease Research Center, Medical University of South Carolina, Charleston, South Carolina, USA

**Keywords:** chronic liver disease, cirrhosis, clinically significant portal hypertension, gastric varices, variceal bleed

## Abstract

The prevalence of liver injury, fibrosis, and, in particular, cirrhosis in the United States is increasing in parallel to the current epidemic of metabolic dysfunction–associated steatotic liver disease and alcohol-associated liver disease. As fibrosis advances, portal hypertension occurs, and when the pressure gradient meets or exceeds 10 mm Hg, the patient is at an increased risk for decompensating events such as esophageal varices. The risk of death also increases. Therefore, decreasing the risk of progression to decompensated cirrhosis is an important management goal. The American Association for the Study of Liver Diseases recently published a guidance document to “coalesce best practice recommendations for the identification of portal hypertension, for prevention of initial hepatic decompensation, for the management of acute variceal hemorrhage, and for reduction of the risk of recurrent variceal hemorrhage in chronic liver disease.” In this updated guidance, the new terms “advanced chronic liver disease” and “clinically significant portal hypertension” have been proposed for routine use in clinical practice. Following recommendations for advanced chronic liver disease identification, which are largely defined by transient elastography measurements of liver stiffness, guidance is provided on the identification of clinically significant portal hypertension and early administration of nonselective beta-blocker therapy in clinically significant portal hypertension for prophylaxis. Optimal control of active bleeding, the role of preemptive TIPS, and gastric varices management are also addressed. Despite the wealth of information provided, the guidance can be difficult to put into practice, leaving non-liver-focused clinicians with an unmet need for a simplified approach to guidelines in general. To address this issue, a panel of hepatologists met to review and discuss the real-world implications of this new guidance and the result is this expert perspective review. This review aims to facilitate improvements in risk stratification and management of variceal bleeding, streamline controversial and complex issues in the recent guidance in a practical way for clinical use, and make recommendations on how to incorporate this important new guidance document into clinical practice.

## Graphical abstract

**Figure:**
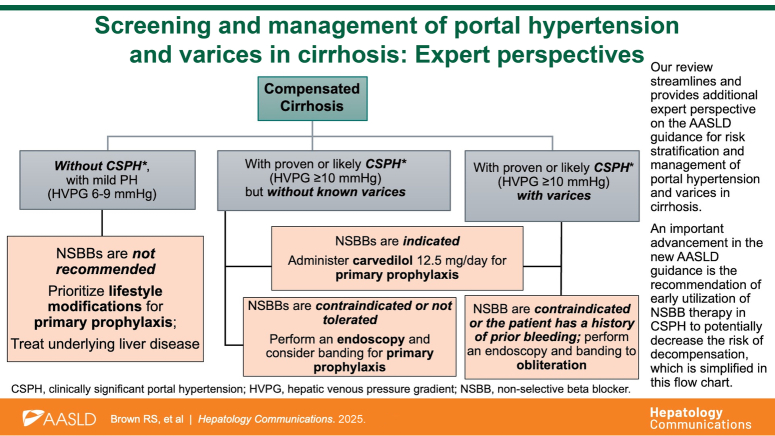

## INTRODUCTION

Advancing cirrhosis leads to disruption of liver architecture and portal hypertension (PH), which reduces portal blood flow and leads to the development of portosystemic collaterals. The HVPG measurement is the gold standard method to evaluate the presence and severity of PH. Decompensation most commonly occurs when HVPG meets or exceeds 10 mm Hg and is thought to increase in likelihood as the HVPG progressively increases. In a study that followed patients for a median of 4 years, patients with an HVPG <10 mm Hg had a 90% probability of not developing clinical decompensation (defined in this study as the development of ascites, variceal hemorrhage, or HE).^1^ The literature indicates that patients in the “high-risk compensated cirrhosis” category, as determined by HVPG, should be considered for early and effective interventions to reduce portal pressure and improve long-term outcomes.^2^ In a study of cirrhotic patients receiving propranolol for prevention of variceal rebleeding, a decrease in HVPG to >20% of baseline or <12 mm Hg is associated with a marked reduction in the long-term risk of developing complications of PH and improved survival. Nonresponders to treatment (n=45) had a significantly greater risk of developing variceal rebleeding (*p*=0.013), ascites (*p*=0.025), spontaneous bacterial peritonitis (*p*=0.003), hepatorenal syndrome (*p*=0.026), and HE (*p*=0.024) than responders (n=28). Eight-year cumulative probability of survival was significantly lower in nonresponders than in responders (52% vs. 95%, respectively, *p*=0.003).^3^ A separate study found that carvedilol has a greater portal hypotensive effect than propranolol in patients with cirrhosis.^4^ Although HVPG is an excellent prognostic tool, it is an invasive test that is infrequently performed in clinical practice, thus non-invasive tests (NITs) that predict elevated HVPG are an unmet medical need.

## APPROACH

The American Association for the Study of Liver Disease (AASLD) recently published 2 complimentary documents that inform practice in the use of noninvasive assessments of PH and should be used to evaluate all patients for the presence of PH.^5^,^6^ One of these AASLD guidance documents addresses the management of varices in cirrhosis.^6^ Practicing clinicians have an unmet need for a simplified approach to guidelines in general. To meet that need, a panel of expert hepatologists in the management of PH and variceal bleeding who are members of or work closely with the Chronic Liver Disease Foundation, a nonprofit 501(c)(3) educational organization dedicated to raising awareness of the liver disease, met to discuss this new guidance. Interactive discussions by this panel of experts focused on the evidence, and recommendations were formulated based on areas of controversy, new recommendations, and areas where data are limited. The result is this expert perspective review, which seeks to facilitate improvements in risk stratification and management of variceal bleeding, streamlines controversial and complex issues in the recent guidance in a practical way for clinical use and recommends how to incorporate this guidance into clinical practice.

## A REVIEW OF UPDATED TERMINOLOGY

Table 1 provides a glossary of new terminology introduced in the AASLD guidance, as well as a review of relevant existing terminology.^7^,^9^ It is important to become familiarized with the newly designated terms advanced chronic liver disease (ACLD) and clinically significant portal hypertension (CSPH) in order to understand and implement the updated recommendations. While cirrhosis is a histologic diagnosis, ACLD is an attempt to confirm advanced fibrosis/cirrhosis in patients based only on NITs. Compensated ACLD (cACLD) is defined per the Baveno VII criteria, which are largely based on transient elastography (TE) measurements of liver stiffness. A liver stiffness measurement (LSM) <10 kPa excludes cACLD, and >15 kPa assumes cACLD.^8^ Once the presence of cACLD (which approximates compensated cirrhosis [CC]) is confirmed, the patient is at risk for CSPH. CSPH is defined as cACLD or CC with an HVPG ≥10 mm Hg and a high likelihood of clinical features of PH, such as varices. CC and cACLD should define the same population of patients in clinical practice. Due to the historic use of compensated cirrhosis, we have chosen the term CC for this review, accepting that it is likely that CC and cACLD are largely interchangeable. Because of the strong association with clinical outcomes, patients with CC should be subclassified during clinical encounters, preferentially using NITs, into those with and without CSPH. Following recommendations for CSPH identification, guidance is provided on the identification of CSPH and early administration of nonselective beta-blocker (NSBB) therapy in CSPH for prophylaxis. This review will also discuss optimal control of active bleeding, the role of preemptive TIPS, and gastric varices (GVs) management.

**TABLE 1:** A glossary of new and updated terminology

Term	Definition
Advanced chronic liver disease (ACLD)^7^	A patient likely to have or close to *having* cirrhosis based on noninvasive measurements (eg, LSM and platelet count) in lieu of histology, clinical features and radiology.
Compensated advanced chronic liver disease (cACLD)^7^,^8^	The term for patients with ACLD without prior decompensation. LSM by TE <10 kPa rules out cACLD and ≥15 kPA rules in cACLD.
Compensated cirrhosis (CC)^7^	The presence of cirrhosis based either on biopsy or the presence of features of cirrhosis, clinically and radiologically, without complications of portal hypertension.
Acute variceal hemorrhage (AVH)	Gastrointestinal bleeding from esophageal or gastric varices.
Clinically significant portal hypertension (CSPH)^5^,^7^	Defined as HVPG ≥10 mm Hg, which has a strong association with clinical outcomes. LSM by TE can be further used to rule in CSPH at values >25 kPa (in patients who are not obese). Additional clinical features that are surrogate markers of CSPH include the presence of gastroesophageal varices on endoscopy and/or portosystemic collaterals on cross-sectional abdominal imaging. Because of the strong association with clinical outcomes, patients with CC/cACLD should be subclassified into those without and with CSPH during clinical encounters preferentially using noninvasive tests.
Decompensation^7^	The development of clinically overt complications of PH (HVPG ≥10 mm Hg), specifically overt ascites, variceal hemorrhage or overt hepatic encephalopathy.
Further decompensation^7^	Patients with decompensated cirrhosis who develop *successive complications* (ie, recurrent variceal hemorrhage, refractory ascites, hepatic encephalopathy, hepatorenal syndrome, spontaneous bacterial peritonitis, hepatic hydrothorax). This new designation was developed because these patients exhibit much higher mortality rates.
Metabolic dysfunction–associated steatohepatitis (MASH),^9^ ^a^	Formerly termed nonalcoholic steatohepatitis (NASH)
Metabolic dysfunction–associated steatotic liver disease (MASLD)^9^ ^a^	Formerly termed nonalcoholic fatty liver disease (NAFLD)
Hepatic venous pressure gradient (HVPG)^7^	The pressure difference between the wedged hepatic vein pressure (which approximates portal vein, venous inflow into the liver, pressure) and the free hepatic vein pressure In healthy participants, normal HVPG is between 1 and 5 mm Hg; PH is defined as a HVPG ≥5 mm Hg; CSPH is defined as HVPG ≥10 mm Hg

^a^ For more information on classifying MASH and MASLD, visit: https://www.aasld.org/new-masld-nomenclature.

Abbreviations: ACLD, advanced chronic liver disease; cALD, compensated advanced chronic liver disease; CC, compensated cirrhosis; CSPH, clinically significant portal hypertension; LSM, liver stiffness measurement; MASH, metabolic dysfunction–associated steatohepatitis; MASLD, metabolic dysfunction-associated steatotic liver disease; PH, portal hypertension; TE, transient elastography.

## WHO TO SCREEN FOR CSPH

The AASLD guidance recommends noninvasive recognition of cACLD as an important quality measure for risk stratification in the care of patients with liver disease. cACLD can likely be measured noninvasively using LSM, which is most commonly done using TE. LSM <10 kPa rules out cACLD with high (but not complete) confidence, while ≥15 kPA rules in cACLD with high confidence. Additional AASLD guidance suggests that CSPH is very likely to be present at an LSM ≥20 kPa and platelets below 150,000/mm^3^ or LSM ≥25 kPa.^6^ Identification of CSPH is important because it is associated with a higher risk of decompensation and patient mortality.^7^ Although HVPG measurement is considered to be the gold standard method to assess portal pressure in patients with cirrhosis, the presence of gastroesophageal varices (GOVs) on endoscopy or portosystemic collaterals or hepatofugal flow on imaging, is also sufficient to diagnose CSPH. The AASLD has also recommended obtaining annual LSM by TE (or non-TE approaches when validated cutoffs exist) and platelet counts in those with cACLD without baseline CSPH, as this provides prognostic information about disease progression.^7^ Many experts use the Rule of Five for LSM, with each increase of 5 kPA yielding prognostic information when combined with platelet count (Table 2).

**TABLE 2:** The Rule of Five for LSM

Criteria	Classification
LSM <10 kPA	No cACLD
LSM 10–14.9 kPA	cACLD without CSPH
LSM 15–19.9 kPA; platelets <110 K/mm^3^	CSPH
LSM 20–24.9 kPA; platelets <150 K/mm^3^	CSPH
LSM ≥25 kPA	CSPH

Abbreviations: cACLD, compensated advanced chronic liver disease; CSPH, clinically significant portal hypertension; LSM, liver stiffness measurement.

## SCREENING METHODS FOR CSPH AND THEIR LIMITATIONS

Although HVPG remains the best way to assess the presence of CSPH, the use of HVPG in clinical practice is impractical. Additionally, HVPG requires expert operators, and its use has been criticized because of heterogeneity and lack of standardization in its performance. There may also be difficulty in interpreting HVPG in certain liver diseases. For example, HVPG may underestimate portal pressure in patients with primary biliary cholangitis, given the component of pre-sinusoidal PH. In patients with cirrhosis related to metabolic dysfunction–associated steatohepatitis, it should be noted that varices and PH signs can develop in patients with HVPG <10 mm Hg. In addition, large shunts can decrease HVPG, thereby causing an underestimation.

TE is also associated with limitations. Not all clinics have the necessary equipment to perform TE, and patients may have to travel a distance to access a clinic with this capability. Furthermore, TE also requires skilled operators and LSM accuracy can be impacted by many factors, as discussed further in Table 3.^7^,^10^ Shear wave elastography may be a more readily available alternative to TE in some settings and likely has the same advantages and limitations, although the cutoffs for shear wave elastography that predict cACLD and CSPH are not well validated. The limitations of TE and other blood and imaging-based NITs in their estimation of liver disease stage, as well as CSPH, are reviewed in detail in Table 3.

**TABLE 3:** Limitations of TE and serum biomarkers in the staging of liver disease and diagnosis of CSPH.^7^,^10^

Tool	Condition	Result
TE/LSM	Ascites Narrow intercostal space Obesity^a^	Failure
	Chronic kidney disease Viral eradication in HCV	Underestimation of the stage of cALD
	Active alcohol use Acute sickle cell crisis Ascites Critical illness Elevated ALT and/or AST (inflammatory hepatitis) Hepatic congestion of cardiac/pulmonary vascular origin Hepatic infiltration Hepatic venous outflow tract obstruction Narrow intercostal space Obstructive cholestasis Postprandial state Sinusoidal obstruction syndrome Steatosis	Overestimation of stage of cALD
APRI	Chronic kidney disease Splenectomy	Underestimation
	Elevated ALT and/or AST (inflammatory hepatitis) Thrombocytopenia not related to PH	Overestimation
FIB-4	Chronic kidney disease Splenectomy	Underestimation
	Elevated ALT and/or AST (inflammatory hepatitis) Thrombocytopenia not related to PH	Overestimation
ELF	Gastrectomy Extrahepatic fibrosing conditions	Overestimation

^a^ TE may be performed successfully in obese patients by using an extra-large probe if the skin-to-liver distance is <25 mm.

Abbreviations: APRI, AST to Platelet Ratio Index; cALD, compensated advanced chronic liver disease; CSPH, clinically significant portal hypertension; ELF, Enhanced Liver Fibrosis test; FIB-4, Fibrosis 4 Index; LSM, liver stiffness measurement; PH, portal hypertension; TE, transient elastography.

Based on the limitations of TE, as highlighted above, the panel suggests further additional recommendations beyond those of the guidance. In lieu of HPVG assessment, patients with an enlarged spleen with portosystemic collaterals or varices can be diagnosed with PH using radiography. Noninvasive liver disease assessments to diagnose CSPH may be used, especially when there are no clinical signs of cirrhosis. Radiography, TE with or without platelet counts (using the criteria delineated by the AASLD), or endoscopy demonstrating varices can also diagnose CSPH. *If clinicians do not have access to TE, or if TE is technically difficult or provides discordant results, a screening upper endoscopy for varices is advised to guide decisions on the use of NSBBs*.

Many factors can influence the ability to obtain reliable TE measurements, of which obesity is the most commonly encountered in clinical practice. Although this can sometimes be overcome using the extra-large probe, in cases when that is not a feasible alternative, NITs (eg, magnetic resonance elastography) may be needed. The presence of ascites confirms CSPH and thus TE is not needed. Other factors that affect the reliability of TE measurements are discussed in Table 3.

## THE RULE-IN/RULE-OUT CRITERIA FOR THE USE OF NSBBS

**FIGURE 1:**
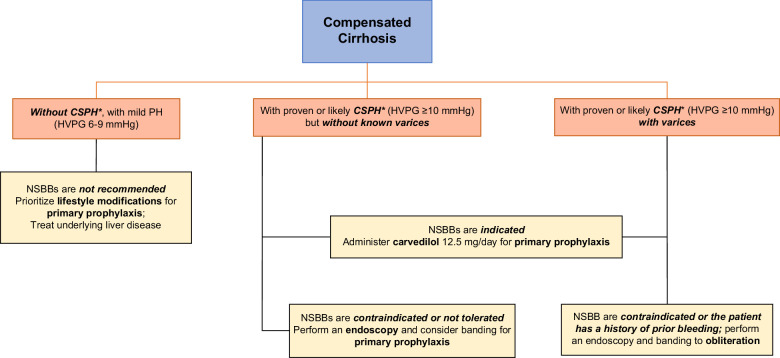
Guidance on the use of NSBBs and screening for and management of AVH in compensated cirrhosis. *Measured by HVPG or estimated by LSM. Abbreviations: AVH, acute variceal hemorrhage; CSPH, clinically significant portal hypertension; LSM, liver stiffness measurement; NSBB, nonselective beta-blocker; PH, portal hypertension.

Figure 1 was created by the panel to streamline the AASLD guidance recommendations and provide additional expert perspectives. An important advancement in the new AASLD guidance is the recommendation for early utilization of NSBB therapy in CSPH to decrease the risk of decompensation.^7^ This section will dissect the flow chart in regard to NSBB recommendations, with specific details provided below.^7^

- *In a patient with CC/cACLD, without CSPH, but with mild PH (HVPG 6–9 mm Hg)*, the AASLD recommends lifestyle modifications and treatment of the underlying liver disease to prevent progression to CSPH and decompensation. Lifestyle modifications in patients with metabolic dysfunction–associated steatohepatitis include weight loss, control of diabetes, control of lipids and alcohol avoidance. NSBBs are not indicated at this time.
- *In a patient with CC with proven or likely CSPH (HVPG >10 mm Hg), but without varices*, the goal is to prevent the development of clinical decompensation. To achieve this goal, the AASLD recommends “the consideration of NSBB administration, with preference for carvedilol 12.5 mg/d.” The panel echoes the importance of achieving this new goal and deems the use of carvedilol a *must* rather than a consideration. In addition to NSBB activity, carvedilol exerts intrinsic anti-alpha-1-adrenergic activity, facilitates the release of nitric oxide and induces intrahepatic vasodilation, further reducing portal pressure. Decreases in HVPG are therefore more pronounced with carvedilol administration compared to other NSBBs.^7^
- *In a patient with CC and proven or likely CSPH (HVPG >10 mm Hg) without acute variceal hemorrhage (AVH)*, prompt administration of carvedilol 12.5 mg/d is necessary for primary prophylaxis of hepatic decompensation, regardless of relative contraindication (eg, asthma) or prior intolerance to NSBBs. If patients are already taking a selective beta-blocker, they should be switched to carvedilol.
- *In a patient with CC and proven or likely CSPH (HVPG >10 mm Hg) with proven AVH*, stabilizing the patient and treatment of the active bleeding is the priority (see section *Control of active bleeding*).
- *In a patient with prior AVH*, administration of carvedilol 12.5 mg/d is necessary for primary prophylaxis of recurrent bleeding and hepatic decompensation, regardless of relative contraindication (eg, asthma) or prior intolerance to NSBBs, in combination with serial band ligation to achieve variceal eradication.

## MANAGEMENT OF CLINICALLY SIGNIFICANT PH

The majority of patients treated with NSBBs will tolerate therapy and achieve a reduction in the risk of bleeding and decompensation. It has been suggested that a relevant proportion of patients do not respond to NSBBs, which raises questions regarding the need for individualized therapy. However, a recent meta-analysis identified 18 studies that included 965 patients. A comparison between beta-blockers and placebo showed a pooled variable ratio of 0.99 (95% CI:0.87–1.14), which suggests a homogeneous HVPG response to NSBB at the individual patient level (ie, no evidence to support that some patients responded to beta-blockers and others did not). For the comparison between carvedilol and propranolol, pooled variable ratio was 0.97 (95% CI 0.82–1.14), suggesting that carvedilol achieves a greater average response, rather than an increase in the proportion of responders. This analysis did not support the existence of a heterogeneous patient-by-patient response to NSBBs in cirrhosis and challenged the concept of personalized therapy based on portal pressure response. These data suggest that routine portal pressure measurement may not be necessary to guide NSBB therapy.^11^

## THE USE OF UPPER ENDOSCOPY

**FIGURE 2:**
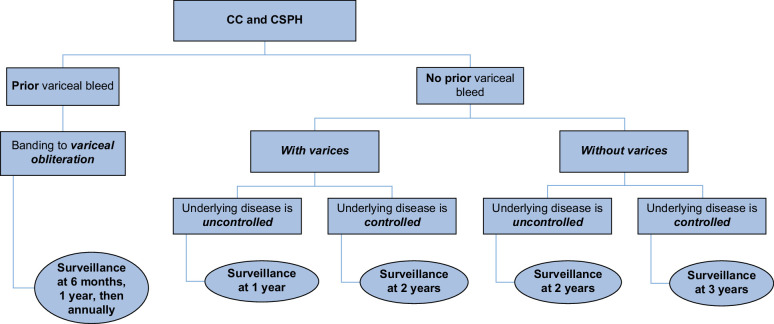
Recommendations for surveillance endoscopy in patients with contraindications/intolerance to NSBBs.^7^ Abbreviations: CC, compensated cirrhosis; CSPH, clinically significant portal hypertension; NSBBs, NSBB, nonselective beta-blockers.

Throughout the guidance, the AASLD provides recommendations on the indications for and use of upper endoscopy for screening, surveillance, and treatment of varices. In most situations, if patients are on an NSBB, they do not need an endoscopy because the results will not change the management strategy (unless prophylactic banding is implemented or acute bleeding is suspected; see section *Control of active bleeding*). However, the following scenarios warrant the use of upper endoscopy:

- *In a patient with suspected CSPH, but TE is not available for screening* (Figure 1), endoscopy is necessary (see Screening methods for CSPH and their limitations?). CSPH may be suspected in patients with thrombocytopenia, or evidence of splenomegaly or portosystemic collaterals on imaging.
- *In a patient with CC with likely CSPH based on NITs (HVPG >10 mm Hg) who has contraindications or is intolerant to NSBBs* (Figure 1), an endoscopy is indicated for variceal surveillance, with intervals depending on the presence or absence of varices on the index endoscopy and whether the underlying disease is controlled (Figure 2).^7^ Examples of NSBB contraindications include severe asthma, advanced heart block, and bradyarrhythmias.^7^ These patients will most likely require prophylactic endoscopic variceal ligation or banding (see sections *Control of active bleeding* and *E* *ndoscopic variceal ligation*) if large esophageal varices (EVs) are present on endoscopy.^7^
- *When AVH is suspected*, the first priority is appropriate resuscitation techniques (see sections *Control of active bleeding* and *Resuscitation*). Use caution when administering fluids to avoid over-resuscitation, and a restrictive blood transfusion approach is recommended.^12^ Additionally, patients with suspected AVH should receive empiric octreotide or terlipressin therapy and broad-spectrum antibiotics. Following stabilization, endoscopy should be performed as soon as possible and no later than 24 hours after presentation. Current guidelines suggest that endoscopy should be performed within 12 hours of presentation in patients with suspected AVH,^7^ although the data supporting that 12 hours is superior to 24 hours are limited.

## CONTROL OF ACTIVE BLEEDING

Control of AVH is further discussed in the subsections that follow.

### Resuscitation

As in all patients with gastrointestinal hemorrhage, the initial approach is to support hemodynamics and ensure that the patient is appropriately resuscitated. Packed red blood cell transfusions should target a hemoglobin of ~7 g/dL,^13^ unless higher targets are required due to comorbid conditions, such as ischemia to end organs. Fresh frozen plasma and platelet transfusions should not be administered based on international normalized ratio (INR) or platelet count targets, respectively, because there is no evidence of benefit of such transfusions in AVH, and in the case of fresh frozen plasma, there is evidence of potential harm. A platelet count above 50,000 or INR <1.5 should not require correction. Fibrinogen measurements may be useful with cryoprecipitate to correct to a level >100 during an episode of active bleeding. It should be emphasized that over-resuscitation is common and detrimental as it will increase portal pressure; thus, close attention to minimizing all supportive fluids is required. The use of thromboelastography to guide the use of platelets, cryoprecipitate, and fresh frozen plasma is useful but is not routinely available in many centers.

### Endoscopic variceal ligation

In the patient with upper gastrointestinal bleeding and medium-to-large (grade 2 or larger) EVs or GVs, the source of bleeding should be presumed to be the varices unless there is another actively bleeding or clearly high-risk lesion identified. In patients with medium-to-large EVs or GVs that are thought to be the source of bleeding, treatment is indicated. For EVs, band ligation (“banding”) is the preferred treatment. Banding should be repeated every 2–4 weeks until obliteration, and then endoscopy repeated at 6 months and then every 12 months to assess for the reappearance of varices requiring additional treatment (Figure 2).^7^ The management of GVs is more complicated, and these patients should be referred to a hepatologist for specialty care (see below).

### Vasoactive therapy

All patients with known or suspected cirrhosis presenting with acute gastrointestinal bleeding should be initiated on vasoactive therapy as soon as possible. There is no U.S. Food and Drug Administration (FDA)-an approved vasoactive drug for varices; therefore, octreotide (initial intravenous [IV] bolus of 50 μg and continued infusion at a rate of 25–50 μg/h) or terlipressin (2 mg IV every 4–6 h for the initial 24–48 h, then 1 mg IV every 4–6 h) are recommended for off-label use since most of the data are based on studies using these drugs.^7^ Octreotide is a somatostatin analog that inhibits the release of glucagon and other vasodilator peptides, leading to vasoconstriction in splanchnic, portal, and systemic circulations and has been the most commonly used drug for this indication in the United States.^14^ Terlipressin is a vasopressin analog that binds to the V1 receptors of vascular smooth muscle cells, leading to vasoconstriction, mainly of the splanchnic circulation.^15^,^16^ The guidance also discusses the vasoactive drug somatostatin for off-label use,^7^ but the panel feels that there is not enough data to support this recommendation.

When administering vasoactive agents, patients should be closely monitored for adverse events. Terlipressin is currently approved in the United States for hepatorenal syndrome-acute kidney injury. In these patients, the most common adverse reactions (≥10%) associated with terlipressin use include abdominal pain, nausea, diarrhea, dyspnea, and respiratory failure. The latter complication has led to FDA-mandated prescribing information, with a boxed warning for serious or fatal respiratory failure; patients with volume overload, including those who have received large volumes of albumin and patients with acute-on-chronic liver failure grade 3, are at increased risk. Assessing oxygenation saturation (eg, SpO_2_) before initiating terlipressin and avoiding administration in patients experiencing hypoxia (eg, SpO_2_ <90%) until oxygenation levels improve is recommended. Patients need monitoring for hypoxia using continuous pulse oximetry during treatment. Terlipressin should be discontinued if SpO_2_ decreases below 90%. Terlipressin is contraindicated in patients experiencing hypoxia or worsening respiratory symptoms and in patients with ongoing coronary, peripheral, or mesenteric ischemia.^17^

With regard to octreotide, repeated boluses in patients with AVH are associated with significant tachyphylaxis.^18^ It is postulated that continuous exposure to somatostatin analogs leads to receptor phosphorylation, G protein uncoupling, receptor internalization, and degradation, resulting in tachyphylaxis.^19^ Tachyphylaxis is not a risk associated with terlipressin, likely because it is vasopressin, not a somatostatin analog. Octreotide used over short periods of time appears to be safe and is not associated with these complications. Patients who do not tolerate octreotide or who continue to bleed despite administration can be switched to terlipressin.

The optimal duration of use for vasoactive agents in AVH is unknown. These drugs should be used cautiously, especially given the risk of tachyphylaxis with octreotide. The panel recommends administering terlipressin or octreotide for no longer than 3 days. When it comes to choosing one drug over another in terms of efficacy, there are no comparative studies. However, since the best currently available treatment for varices appears to be band ligation, it is likely that any differences in the effectiveness of the 2 vasoactive agents would be difficult to ascertain. It should be noted that in Europe, terlipressin remains the standard of care for AVH. In the United States, the cost of terlipressin may deter hospitals from supporting off-label use for AVH.

### Antimicrobial therapy

IV antibiotic treatment should be administered in all patients with suspected or documented CSPH presenting with upper gastrointestinal bleeding, tailored to local resistance patterns and patient allergies. Trials on this topic are scarce. A 2024 Cochrane review analyzed 12 trials (n=1241) evaluating antibiotic prophylaxis against placebo or no antibiotic prophylaxis and demonstrated clear benefits. Antibiotic prophylaxis was associated with reduced mortality (RR [relative risk], 0.79; 95% CI, 0.63–0.98), mortality from bacterial infections (RR, 0.43; 95% CI, 0.19–0.97), bacterial infections (RR, 0.35; 95% CI, 0.26–0.47), rebleeding (RR, 0.53; 95% CI, 0.38–0.74), and days of hospitalization (MD [mean difference], –1.91 d; 95% CI, –3.80 to –0.02).^20^ The most commonly used agent is ceftriaxone, 1 g/24 h for up to 5 days, but amoxicillin clavulanate and fluoroquinolones have also been used. Patients can be switched to an oral antibiotic once they are able to tolerate a regular diet to complete a 5-day course. Antimicrobial therapy may also be discontinued once bleeding is controlled and in the absence of an active infection,^7^ but most patients should receive a full 5-day course of antibiotics.

### Additional considerations

Enteral feeding should be started once the AVH episode has been controlled. It should also be noted that the presence of variceal bands does not contraindicate the placement of a feeding tube if indicated. Proton pump inhibitors should be discontinued once AVH has been confirmed as the bleeding source in the absence of other specific indications.^7^

## PREEMPTIVE TIPS

The role of “early” or preemptive TIPS in specific patients with AVH considered to be high risk is controversial. A landmark study demonstrated its benefit in patients with Child–Pugh (CP) class B or C.^21^ AASLD guidance recommends TIPS within 24–72 hours of the initial endoscopy in these patients.^7^ However, data supporting early TIPS were generated in highly selected patients, and a wide variety of patients were excluded from studies evaluating the safety and efficacy of early TIPS. Given the complexity of decision-making for patients with CP class B scores >7 with active bleeding and CP class C scores 10–13 with and without transplant consideration, the panel stresses the need for input and guidance from an expert hepatologist or gastroenterologist with expertise in PH and critical care hepatology. Hepatic dysfunction and encephalopathy may worsen with TIPS, and hepatology input is critical in decision-making, especially if transplantation is a consideration. Thus, there is no established cutoff for MELD/CP where TIPS should be universally used. Rather, a case-by-case discussion should occur, balancing the risk of liver failure and HE post TIPS with the potential benefits. Transplant candidacy should also be considered as TIPS in this situation is likely to be most effective as a bridge to potential liver transplant. Given the expertise required for placement and the risk of complications, the panel recommends TIPS placement only by individuals with significant technical expertise in the procedure. In patients with uncontrolled AVH, the use of either balloon tamponade or covered expandable esophageal stents as a bridge to TIPS or transplantation is likely the safest option.^7^

### TIPS and the risk of HE

In a study from Germany, in-hospital mortality of cirrhotic patients with TIPS decreased when it was placed for severe bleeding (15.2% [TIPS] vs. 19.5% [endoscopy treatment]); ascites (8.7% [TIPS] vs. 14.4% [paracentesis]); and hepatorenal syndrome (17.1% [TIPS] vs. 43.3% [no TIPS]). However, during hospitalization, 22.6% of the admissions of patients with TIPS insertion showed HE. In the subgroup analyses, in-hospital mortality in patients admitted with HE grades 1 or 2, and TIPS was lower than in patients without TIPS. In the logistic regression, a higher HE grade (3 and 4), infection, and circulatory disease were found to be independently associated with in-hospital mortality in patients with TIPS insertion.^22^ One example of a high risk of HE occurrence is if the portosystemic gradient is reduced by >60% after TIPS placement, then TIPS-related refractory HE can occur, and prophylactic therapy for HE should be considered. Several therapies have been studied to prevent HE in patients undergoing TIPS. The panel recommends consideration of prophylaxis with lactulose and/or rifaximin in select patients deemed at increased risk for HE following TIPS.

## GV MANAGEMENT

The diagnosis and management of GVs is often challenging. All patients with GVs should be considered to have CSPH and should be administered NSBBs for primary prophylaxis. However, because GVs bleed at a lower pressure (sometimes 6 or 8 mm Hg), not all patients with GVs will have measurable CSPH due to shunted blood. In these patients, the efficacy of NSBBs is unclear. The guidance recommends that patients with high-risk cardio fundal varices (GOV type 2 or isolated GV type 1) ≥10 mm, red wale signs, CP class B/C, and who have contraindications or intolerance to NSBBs should be considered for primary prophylaxis with endoscopic cyanoacrylate injection.^7^ The panel stresses that cyanoacrylate administration should only be performed in an experienced center and in high-risk cases with appropriate informed consent.

For GV with bleeding, endoscopic examination may be difficult or inconclusive due to excessive bleeding and pooling of the blood in the gastric fundus, potentially obscuring even large GV. In the setting of any question about the presence of GVs, for example, if blood cannot be cleared from the stomach, a repeat endoscopy should be considered within 24 hours to evaluate for GVs or larger EVs with or without a prokinetic agent like erythromycin. Once GVs are identified, cross-sectional contrast imaging should be considered in all patients to evaluate for splenic and PVT. Initial management of bleeding GVs should be identical to the management of bleeding EVs, including vasoactive therapy, antimicrobials, conservative transfusion strategy, and endoscopic evaluation, within 12 hours.^7^ Cyanoacrylate injection or endoscopic ultrasound–guided coils for obliteration are usually more successful than band ligation for GV with bleeding. TIPs or balloon-occluded retrograde transvenous obliteration should *not* be used for bleeding prophylaxis for GV^7^ but can be effective for the treatment of GV with bleeding. Since the vast majority of both GV (and ectopic varices) bleed with low portal pressure (due to effective decompression by the variceal collateral such as a spontaneous splenorenal shunt for proximal GV), both embolization (to occlude the collateral) by balloon-occluded retrograde transvenous obliteration (BRTO) or Coil-Assisted Retrograde Transvenous Obliteration (CARTO) and/or TIPS placement (to prevent complications such as recurrent ectopic variceal bleeding and/or ascites by preventing re-creation of severe PH after shunt occlusion) is advisable.

## SECONDARY PROPHYLAXIS

Survivors of an episode of active variceal bleeding have a 60%–70% risk of recurrent bleeding within 1 year.^23^ Secondary prophylaxis should be initiated immediately after control of the first bleed (Figure 1).^7^ All patients should be administered an NSBB, unless a patient has absolute contraindications or does not tolerate the NSBB, with carvedilol remaining the NSBB of choice.^7^ In all patients, follow-up esophagogastroduodenoscopy with potential further band ligation is recommended to achieve or confirm eradication of EVs and to assess for, as a baseline, GVs, severe PH gastropathy, or gastric antral vascular ectasia syndrome at the intervals shown in Figure 2.

## FUTURE DIRECTIONS

The updated guidance and associated terminology represent an attempt to standardize terminology, harmonize with current clinical practice, and improve utilization of treatments aimed at preventing hepatic decompensation and preventing and treating AVH, but progress is still needed in this field. Widespread access to TE is needed and is currently suboptimal due to cost and logistics. Additional NITs, such as ultrasound with shear wave elastography or abbreviated magnetic resonance elastography, may become cost-effective options for diagnosing CSPH while performing screening for HCC. The benefits of NSBB therapy for prophylaxis are clear, but many questions remain. It is still controversial when or if to stop NSBB treatment in the setting of decompensation, particularly with refractory ascites and renal dysfunction, and this is not addressed by the current guidelines. Another question is whether treatment or cure of the underlying cause of cirrhosis can reverse CSPH and eliminate the need for NSBBs in select patients. Are there other agents that have similar disease-modulating effects, such as statins? The role of early TIPS also remains controversial. Though early TIPS is likely underused, it is a permanent, usually irreversible, intervention. Although rare, refractory HE, even at a low MELD score, can become a significant issue after TIPS, with limited access to transplant and few management options. Further research in this field can only improve risk reduction prevention and management strategies.
